# Demographic trends in the incidence of malignant appendiceal tumours in England between 1995 and 2016: Population-based analysis

**DOI:** 10.1093/bjsopen/zrac103

**Published:** 2022-08-27

**Authors:** Philippa Orchard, Ryan Preece, Michael G Thomas, Steven W Dixon, Newton A C S Wong, Adam C Chambers, David E Messenger

**Affiliations:** University Hospitals Bristol and Weston NHS Foundation Trust, Bristol, UK; University Hospitals Bristol and Weston NHS Foundation Trust, Bristol, UK; University Hospitals Bristol and Weston NHS Foundation Trust, Bristol, UK; School of Cellular and Molecular Medicine, University of Bristol, Bristol, UK; Department of Cellular Pathology, North Bristol NHS Trust, Southmead Hospital, Bristol, UK; School of Cellular and Molecular Medicine, University of Bristol, Bristol, UK; University Hospitals Bristol and Weston NHS Foundation Trust, Bristol, UK

## Abstract

**Aims:**

Recent data suggest that the incidence of malignant appendiceal tumours is increasing. This study aimed to determine temporal trends in the incidence of malignant appendiceal tumours within England and a possible influence by demographic factors.

**Methods:**

All incident cases of appendiceal tumours in patients aged 20 years and above were identified from the National Cancer Registration and Analysis Service database between 1995 and 2016 using ICD-9/10 codes. Cancers were categorized according to histology. Joinpoint regression analysis was used to investigate changes in age-standardized incidence rates by age, sex, histological subtype and index of multiple deprivation quintiles, based on socioeconomic domains (income, employment, education, health, crime, barriers to housing and services and living environment). Average annual per cent changes (AAPCs) were estimated by performing Monte-Carlo permutation analysis.

**Results:**

A total of 7333 tumours were diagnosed and 7056 patients were analysed, comprising 3850 (54.6 per cent) neuroendocrine tumours (NETs), 1892 (26.8 per cent) mucinous adenocarcinomas and 1314 (18.6 per cent) adenocarcinoma (not otherwise specified). The overall incidence of appendiceal tumours increased from 0.3 per 100 000 to 1.6 per 100 000 over the study interval. Incidence rate increases of comparable magnitude were observed across all age groups, but the AAPC was highest among patients aged 20–29 years (15.6 per cent, 95 per cent c.i 12.7–18.6 per cent) and 30–39 years (14.2 per cent, 12.2–16.2 per cent) and lowest among those aged 70–79 years (6.8 per cent, 5.7–8.0 per cent). Similar incidence rate increases were reported across all socioeconomic deprivation quintiles and in both sexes. Analysis by grade of NET showed that grade 1 tumours accounted for 63 per cent between 2010 and 2013, compared with 2 per cent between 2000 and 2003.

**Conclusions:**

The incidence rate of malignant appendiceal tumours has increased significantly since 1995 and is mainly attributed to an increase in NETs. The increased diagnosis of low-grade NETs may in part be due to changes in pathological classification systems.

## Teaser

North American studies have shown that appendiceal tumours are increasing in all histological subtypes and ages groups. This paper is the first to show this in a European population and is important given the current debate regarding conservative management of appendicitis.

## Introduction

Malignant tumours of the appendix are rare neoplasms that often have a non-specific presentation and are most commonly detected as an incidental finding at appendicectomy (approximately 1 per cent of all appendicectomies) or other colonic resections, making them an important consideration for emergency surgical teams^1^. Recent studies suggest that the incidence of malignant appendiceal tumours may be increasing. Two previous studies based on data from North American populations have reported an increase in all histological subtypes of malignant appendiceal tumours. A report from the USA showed a 54 per cent increase in overall incidence (0.63 to 0.97 per 100 000) of appendiceal cancers among a US population from 2000 to 2009^2^. Incidence rate increases were consistent across all histological subtypes, age groups and sexes. This finding was confirmed in a subsequent US–Canadian analysis that demonstrated an overall incidence rate increase of 232 per cent between 1992 and 2016 in the absence of a concomitant increase in the appendicectomy rate^3^. The study showed that the highest incidence rate increase was noted among young patients (15–49 years) with neuroendocrine tumours (NETs). Within Europe this phenomenon has not been demonstrated; a report from Sweden showed an increase in appendiceal adenocarcinomas (2.6 to 5.4 per 1 000 000 between 1970–79 and 2010–12) but this trend was not confirmed in appendiceal neuroendocrine neoplasms^4^. One study from the Netherlands that specifically focused on mucinous appendiceal neoplasms showed a modest increase from 1980 to 2010 that was similar in magnitude in both men and women^5^. While primary appendiceal tumours remain a rare entity, any increase in their incidence should give cause for concern given the unclear aetiology and associated morbidity and mortality. In a multicentre German study of outcomes from appendiceal tumours, a 5-year overall survival of 80 per cent and 50 per cent was reported for appendiceal NETs and epithelial-origin tumours respectively^6^. At the present time, no studies have investigated the incidence rates of primary appendiceal tumour histological subtypes outside North America. Furthermore, an analysis of the impact of socioeconomic status on incidence rates has not previously been undertaken. Therefore, this study aimed to determine temporal trends in the incidence of primary appendiceal tumours within England and the association between such trends and age, sex, socioeconomic status and histological subtype.

## Methods

This study was reported according to the STROBE guidelines for epidemiological studies^7^. The National Cancer Registration and Analysis Service (NCRAS) database (ODR1718_067/A1) was searched to identify all patients older than 20 years of age diagnosed with a primary appendiceal tumour in England between 1995 and 2016. NCRAS collects data on all patients diagnosed with cancer in England to monitor incidence trends and survival, as well to assist in the identification of risk factors and cancer clusters^8^.

### Case identification

Malignant appendiceal cancers diagnosed between 1995 and 2016 were identified using the C18.1 ICD10 code from the NCRAS database. SNOMED morphology codes (International Classification of Diseases for Oncology, Third Edition (ICD-O-3)) were used to subdivide invasive tumours based on the C18.1 anatomical location into histological subgroups: NETs (codes 8240–8249); adenocarcinomas including adenocarcinomas, not otherwise specified (NOS) (8140–8213, 8261 and 8263); and cystic, mucinous and serous neoplasms (8440–8490) (while these tumours are within the overall adenocarcinoma classification they are subdivided from adenocarcinomas, including adenocarcinomas-NOS). Those coded as uncertain or borderline malignant tumours (8000, 8001, 8010 and 8020) were excluded given the inability to confirm morphological subtype. Low-grade appendiceal mucinous neoplasms (LAMNs) were excluded from this analysis as they have a non-invasive SNOMED classification. Tumours were grouped into age bands at diagnosis using a 10-year interval from age 20 years onwards.

### Number of appendicectomies

To determine whether the incidence rate of primary appendiceal tumours was associated with temporal changes in the appendicectomy rate, the number of appendicectomies performed annually in patients aged 15 years and older in the English National Health Service (NHS) was extracted from Hospital Episode Statistics data via the NHS Digital’s Hospital Admitted Patient Care Activity database from 1999 to 2016. This also allowed for a crude estimation of the incidence of primary appendiceal tumours per appendicectomy, based on the assumption that all tumour diagnoses were made at appendicectomy.

### Statistical analysis

Mid-year population estimates were obtained from the Office for National Statistics (ONS) to provide population data stratified by age^9^. Age-specific and age-standardized cancer incidence rates were calculated as previously described^10^. In brief, age-specific incidence rates per 100 000 were calculated for each age group using the mid-year ONS population estimates. Age-standardized incidence rates were then calculated using the European Standard Population (ESP) 2013 in accordance with the method for direct standardization used by the ONS^9^. Cases were further stratified by histological subtype, sex, and index of multiple deprivation (IMD) quintile (from 2001 onwards, as IMD data was incomplete before this) obtained from NCRAS for each patient. IMD is an areas-based calculation using seven weighted domains (income, employment, education, health, crime, barriers to housing and services and living environment). Geographical areas termed lower-layer super output areas are ranked and split into five equal groups (each resulting quintile having 20 per cent of the ranked areas) based on these domains from most to least deprived. Temporal changes in age-specific incidence rates were analysed using Joinpoint Regression Program version 4.9.0.0 (National Cancer Institute)^11^. These were stratified according to histological subtype, sex and IMD. Annual per cent changes (APCs) were estimated by performing Monte-Carlo permutation analysis of the log-transformed age-specific incidence rate to fit a series of joined lines with a minimum of zero and a maximum of five joinpoints. The best fit model was then identified by performing a series of comparisons among fitted models ranging from zero to five joinpoints. The goodness of fit of the joinpoint regression models was estimated by calculating the squared correlation coefficient (*r*^2^) to indicate the extent of agreement between modelled and observed values.

## Results

A total of 7333 patients with primary appendiceal tumours were identified, of which 277 patients were excluded due to an inability to determine their tumour morphology from the SNOMED codes. Therefore, 7056 patients were included in the analysis. There were 3850 NETs (54.6 per cent), 1314 adenocarcinomas, including adenocarcinoma-NOS (18.6 per cent) and 1892 cystic, mucinous and serous neoplasms (26.8 per cent). The cohort consisted of 4589 patients (65.0 per cent) aged older than 50 years and 4011 (56.8 per cent) females. In terms of age distribution by histological subtype, 2579 of 3206 patients (80.4 per cent) diagnosed with adenocarcinoma (cystic mucinous and serous neoplasms and adenocarcinomas, including adenocarcinoma-NOS) were aged older than 50 years, compared with 2010 of 3850 patients (52.2 per cent) with NETs (*Table 1*). Complete data on incidence rates, APC and average APC (AAPC) stratified for age, sex, tumour type and IMD quintile are provided in *Table S1*. Also, the number of appendiceal neoplasms diagnosed across different UK geographical locations has been provided in *Table S2*.

**Table 1.** Population demographics

	Overall (*n* = 7056)	NETs (*n* = 3850, 54.6%)	Adenocarcinoma-NOS (*n* = 1314, 18.6%)	Cystic, mucinous and serous adenocarcinoma (*n* = 1892, 26.8%)
**Number of diagnoses/years**				
1995–2001	949 (13.4)	397 (10.3)	241 (18.3)	311 (16.4)
2002–2009	1975 (28.0)	848 (22.0)	390 (29.7)	737 (39.0)
2010–2016	4132 (58.6)	2605 (67.7)	683 (52.0)	844 (44.6)
**Age (years)**				
20–49	2467 (34.9)	1840 (47.8)	221 (16.8)	406 (21.5)
50+	4589 (65.0)	2010 (52.2)	1093 (83.2)	1486 (78.5)
**Sex ratio (M:F)**	3045 (43.2):4011 (56.8)	1650 (42.9):2200 (57.1)	1650 (42.9):2200 (57.1)	1650 (42.9):2200 (57.1)
**Ethnicity**				
Asian	142 (2.0)	71 (1.8)	41 (3.1)	30 (1.6)
Black	92 (1.3)	50 (1.3)	18 (1.4)	24 (1.3)
Mixed	28 (0.4)	16 (0.4)	3 (0.2)	9 (0.5)
White	6156 (87.2)	3386 (87.9)	1112 (85.6)	1658 (87.6)
Other	79 (1.1)	42 (1.1)	12 (0.9)	25 (1.3)
Unknown	559 (7.9)	285 (7.4)	128 (9.7)	146 (7.7)
**Index of multiple deprivation**				
1 (least deprived)	1376 (19.5)	714 (18.5)	251 (19.1)	418 (22.1)
2	1478 (20.9)	809 (21.0)	265 (20.2)	366 (19.3)
3	1400 (19.8)	769 (20.0)	225 (17.1)	323 (17.1)
4	1217 (17.2)	669 (17.4)	264 (20.1)	398 (21.0)
5 (most deprived)	1095 (15.5)	693 (18.0)	172 (13.1)	230 (12.2)
Unknown	490 (6.9)	196 (5.1)	137 (10.4)	157 (8.3)

Values are *n* (%). NOS, not otherwise specified; NET, neuroendocrine tumour.

### Age-standardized trends in overall incidence according to age, sex and socioeconomic status

**Fig. 1:**
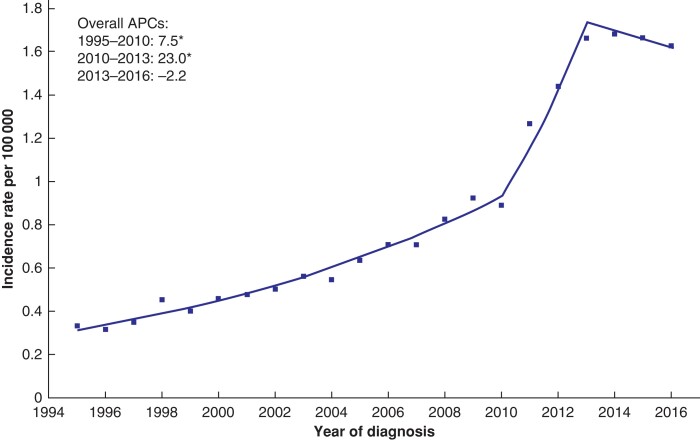
Overall age-standardized incidence of appendiceal tumours 1995–2016 Plotted lines represent the annual percentage change (APC). Asterisk denotes APC that is significantly different from 0 (*P* < 0.050) using the permutation model of logarithmically transformed data.

**Fig. 2:**
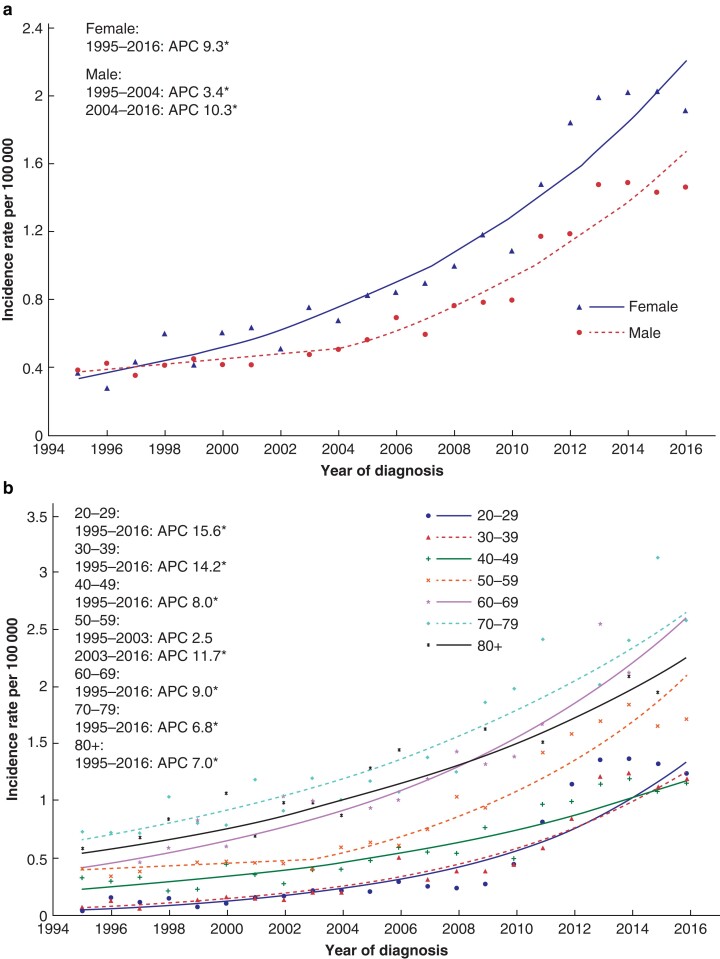
Age-standardized incidence rates of appendiceal tumours **a** Sexes. **b** Age groups. Plotted lines represent the annual percentage change (APC). Asterisk denotes APC that is significantly different from 0 (*P* < 0.050) using the permutation model of logarithmically transformed data.

Overall, the incidence of appendiceal cancers increased from 0.3 to 1.6 per 100 000 between 1995 and 2016. The AAPC was 8.1 per cent (95 per cent c.i. 5.1 to 11.2 per cent). The APC was initially 7.5 per cent (6.6 to 8.4 per cent) from 1995 to 2010 and then increased to 23.0 per cent (1.8 to 48.7 per cent) until 2013 at which point the APC plateaued at −2.2 per cent (−11.0 to 7.5 per cent) (*Fig. 1*). Over the study interval, the AAPC was similar for males (7.3 per cent, 5.6 to 9.1 per cent) and females (9.3 per cent, 8.2 to 10.5 per cent) (*Fig. 2*). While the incidence rate increased across all age groups, the AAPC was highest among those patients aged 20–29 years (15.6 per cent, 12.7 to 18.6 per cent) and 30–39 years (14.2 per cent, 2.2 to 16.2 per cent) and lowest among those aged 70–79 years (6.8 per cent, 5.7 to 8.0 per cent) (*Fig. 2*). Stratification of the study population according to IMD quintile revealed similar incidence rate increases across all quintiles (*Table S1*).

### Age-standardized trends according to tumour morphology

**Fig. 3:**
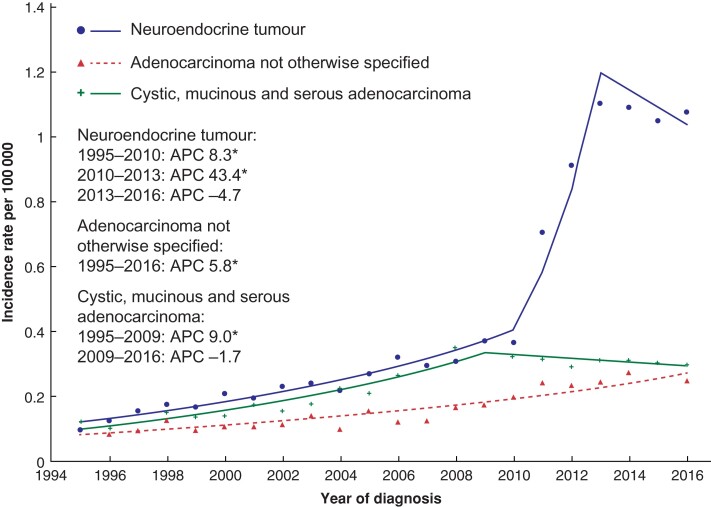
Age-standardized incidence rates of appendiceal tumours by morphology Plotted lines represent the annual percentage change (APC). Asterisk denotes APC that is significantly different from 0 (*P* < 0.050) using the permutation model of logarithmically transformed data.

**Fig. 4:**
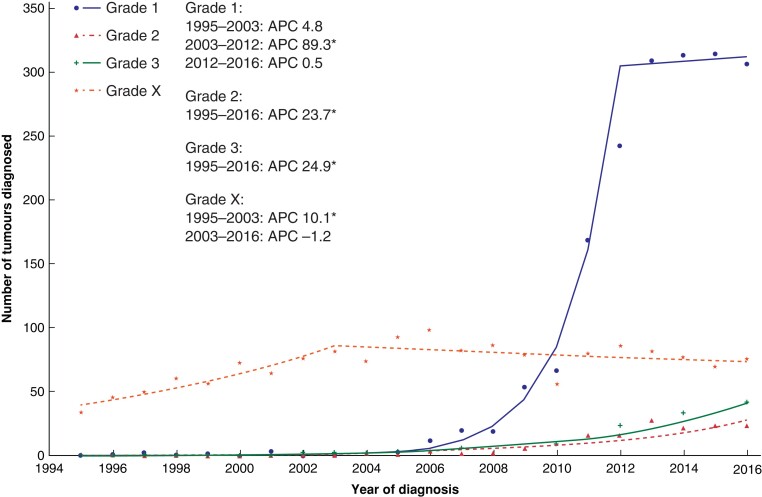
Number of neuroendocrine tumours diagnosed each year by tumour grade Plotted lines represent the annual percentage change (APC). Asterisk denotes APC that is significantly different from 0 (*P* < 0.050) using the permutation model of logarithmically transformed data. Grade X, grade could not be evaluated.

When incidence trends were stratified according to tumour morphology, the largest increase was observed with NETs with an overall rise in incidence from 0.10 to 1.08 per 100 000 population over the study interval. From 1995 to 2010, there was an initial moderate increase in APC of 8.3 per cent (95 per cent c.i. 6.9 to 9.7 per cent), which increased further to 43.4 per cent (5.8 to 94.4 per cent) until 2013, before plateauing at −4.7 per cent (−18.2 to 10.9 per cent). The overall AAPC for NETs between 1995 and 2016 was 10.7 per cent (5.8 to 15.8 per cent) (*Fig. 3*). Patients with adenocarcinomas (cystic mucinous and serous neoplasms or adenocarcinomas, including adenocarcinoma-NOS) were then analysed, with a more gradual increase in incidence noted over the study interval. AAPCs of 5.3 per cent (95 per cent c.i. 3.7 to 7.0 per cent) and 5.8 per cent (4.6 to 7.0 per cent) for cystic, mucinous serous neoplasms and adenocarcinomas, including adenocarcinoma-NOS were observed respectively. A subgroup analysis of the NET cohort was undertaken to determine which factors contributed to the marked increase in incidence between 2010 and 2013. Incidence rate increases for NETs were similar when stratified by both age and sex (*Table S1*). Analysis by grade of NET showed that the main contributory factor to the incidence rate increase between 2010 and 2013 was an exponential increase in the diagnosis of grade 1 tumours (*Fig. 4*). Of all NETs diagnosed between 2010 and 2013, grade 1 tumours accounted for 63 per cent, compared with 2 per cent between 2000 and 2003. From 2003 to 2012, the APC for grade 1 NETs was 89.3 per cent (95 per cent c.i. 58.0 to 126.9 per cent). Incidence rate increases were also noted for grade 2 and 3 NETs with APCs between 1995 and 2016 of 23.7 per cent (19.4 to 28.2 per cent) and 24.9 per cent (20.9 to 29.0 per cent) respectively.

### Appendicectomy rates

Between 1999 and 2016, the number of appendicectomies performed in England per year rose from 30 227 to 40 106. After accounting for the concomitant increase in the English population over this time, the appendicectomy rate increased from 83 per 100 000 to 95 per 100 000 with an AAPC of 0.8 per cent (95 per cent c.i. 0.2 to 1.4 per cent). Based on the assumption that all primary appendiceal tumours were diagnosed at appendicectomy, this corresponded to an increase in the crude incidence of malignancy at appendicectomy from 0.4 per cent in 1999 to 2.0 per cent in 2016.

## Discussion

This large national population study confirms the findings from previous North American studies that the incidence of appendiceal tumours is increasing in all histological subtypes of malignant appendiceal tumours^2,3^. The overall incidence in England rose from 0.3 to 1.6 per 100 000 over the 21-year study interval and incidence rate increases were of similar magnitude across all age groups, sexes and IMD quintiles. While the incidence rate of all histological subtypes of primary appendiceal tumours have increased, the most pronounced incidence rate increase was noted among NETs. Since 2003, grade 1 NETs have been responsible for most of the incidence rise.

The primary appendiceal tumour incidence rates from the present study are similar to those reported in North American populations^2,3^. For example, a past study identified an incidence rate 0.97 per 100 000 population in 2009 from a retrospective analysis of the Surveillance, Epidemiology, and End Results database^2^. The increasing incidence of primary appendiceal tumours warrants consideration of the underlying factors driving this phenomenon. The lack of association between incidence rate trends and age, or sex, observed in the present study is consistent with what has been reported previously^3^. In addition, no association with socioeconomic deprivation and incidence rate trends could be established in this study, which is a new finding. One obvious explanation for the increasing incidence of primary appendiceal tumours is whether these tumours are being detected more frequently by virtue of a concomitant increase in the number of appendicectomies being undertaken. Another investigation suggested that the increase in appendiceal neuroendocrine tumours they observed was probably related to a higher frequency of appendicectomies occurring in the young^4^; however, only a very slight increase in the appendicectomy rate was observed in this study with an AAPC of 0.8 per cent. Thus, appendicectomy rates are unlikely to explain the much higher rise in malignancy incidence rate and echoes the findings of Singh *et al*.^3^. Further, it is not possible to determine whether other resections such as right hemicolectomy or ileocaecectomy contribute to the numbers of appendiceal tumours diagnosed.

A second, and more likely, explanation may be due to changes in pathological assessment methods. The authors are unaware of any formal guidelines on whether all resected appendices should be submitted for histological examination. One study suggested they should not routinely be sent after appendicectomy unless there is an obviously macroscopic abnormality at the time of surgery^12^. This, however, is in contrast with the practice of routine histological examination^13,14^. A recent retrospective study of appendicectomy specimens submitted to an American medical centre suggested that more extensive pathological analysis—with a greater number of representative sections submitted per case—may be contributing to rising incidence rates of appendiceal neoplasms^15^. The Royal College of Pathologists data set for appendiceal carcinomas and mucinous neoplasms recommends processing the entire appendix if an appendiceal neoplasm is encountered or suspected, but this resource was only first published in 2021^16^. Of potentially more relevance are the Royal College of Pathologists tissue pathways that provide detailed guidance on the pathological assessment of routine appendicectomy specimens^17^. The first of these tissue pathways was published in 2009, which is just before the sharper rise in reported incidence of NETs. In the past 20 years, there have also been significant changes to the international pathological classification of digestive tract tumours, particularly for NETs. The 2000/2004 WHO Classification of Tumours of the Digestive System introduced a new classification system for gastrointestinal NETs (well differentiated endocrine tumours, well differentiated endocrine carcinoma and poorly differentiated endocrine carcinoma (PDEC)), which was thought to have introduced confusion and led to poor acceptance and coding practices among pathologists^18,19^. The subsequent 2010 edition simplified NET classification (grade 1 NET, grade 2 NET and neuroendocrine carcinoma) and resulted in most tumours previously classified as carcinoids now being classified as grade 1 NETs^20^. These changes to NET classification probably account for the incidence rate increase in grade 1 NETs observed in the present study: with an initial slow increase from 2003 as pathologists slowly began adopting the WHO 2000/2004 edition and then a more rapid increase from 2010 to 2013 when the 2010 edition was introduced. The plateauing in rates after 2013 likely reflect the widespread adoption of the 2010 edition in reporting practice. The WHO classification underwent its latest iteration in 2019^21^. The resulting changes to the classification of appendiceal neoplasms include introducing a grade 3 for NETs, creating the new category of high-grade appendiceal mucinous neoplasm, and renaming goblet cell carcinoid as goblet cell adenocarcinoma; however, this 2019 publication would not have affected the data collected for the study presented above.

It is known that the incidence of colorectal adenocarcinomas is increasing in the young adult population^10^, although the exact reason for this is unclear and is likely to be multifactorial with both environmental exposure and underlying genetic susceptibility playing a role. Incidence rate increases of appendiceal tumours of all subtypes were also noted in older adults and are unlikely to be explained by the introduction of the national bowel cancer screening programme, as appendiceal tumours are rarely identified at colonoscopy. It is conceivable that the increased use of cross-sectional imaging in contemporary practice may lead to more frequent detection of appendiceal tumours, although a recent study showed that more than 80 per cent of patients with appendiceal tumours presented as either appendicitis or with an abdominal mass^10,22^. Another consideration for the increase in primary appendiceal adenocarcinomas relates to the classification of LAMNs. Before 2010, LAMNs did not have a specific ICD-10 code^20^, and therefore, may have been coded just as adenomas. This changed in 2010 when LAMN was given an ICD-10 code under the adenocarcinoma umbrella (8480); however, this remains a benign behavioural code 8480/1 and so is not included in this analysis^20^. Additionally, incidence rates for adenocarcinomas were already increasing before 2010 and so for these reasons the observed change in incidence would not be explained by the WHO reclassification of LAMNs.

If it is assumed that all the appendiceal tumours in the present study were diagnosed at appendicectomy, then the crude incidence of appendiceal malignancy at operation in 2016 was 2.0 per cent and is comparable to appendiceal malignancy incidence rates reported in recent cohort studies^23,24^. The appendiceal malignancy rate should be an important consideration when deciding on how to manage patients with appendicitis and has received renewed focus during the COVID-19 pandemic as conservative treatment strategies were more frequently adopted. In a recent large prospective observational study of appendicitis management conducted during the COVID-19 pandemic, 36 of 1974 (1.8 per cent) of patients who underwent initial operative management had tumours, compared with 7 of 277 (2.5 per cent) of patients who underwent surgery after initial failed conservative management^23^. The prediction of which patients with appendicitis harbour an appendiceal tumour is not straightforward. Certainly, those with peri-appendiceal abscesses harbour a high rate of tumours and a recent trial comparing interval appendicectomy with MRI follow-up in patients treated conservatively was terminated prematurely owing to ethical concerns regarding a malignancy rate of 20 per cent^25^. While malignancy risk calculators do exist in the setting of appendicitis, these are yet to be externally validated^24^. Currently, the World Society of Emergency Surgery provides a weak recommendation based on low-quality evidence that patients aged 40 years or older with non-operatively managed appendicitis should undergo colonoscopy and interval CT^26^. Long-term follow-up of patients from recent studies will provide important data regarding the late presentation of appendiceal tumours in patients with conservatively managed appendicitis^23^.

This study used the national NCRAS database, which provides near 100 per cent data completeness; however, the study does have several limitations. Stage-specific data for all histological subtypes were not recorded routinely by NCRAS until 2012, preventing further analysis by tumour stage. Certain patient-level data were also unavailable, such as BMI, and thus their association with incidence rates could not be explored. Furthermore, the route to diagnosis, such as emergency *versus* elective presentation, was not recorded, thereby preventing a more accurate estimate of the incidence of malignancy at appendicectomy. Finally, IMD quintile is a group-level metric and does not account for individual-level contextual factors that may have influenced its association with incidence rates.

Overall, this study has confirmed that the incidence of primary appendiceal tumours is increasing and can mainly be attributed to the marked increase in incidence of NETs. Changes to pathological classification systems have undoubtedly had the greatest impact on NET incidence rates, but the increase in adenocarcinomas suggests that other environmental and genetic factors may also play a role. Primary appendiceal tumours are present in approximately 2 per cent of all appendicectomy specimens and clinicians should consider this risk when counselling patients about non-operative management strategies for appendicitis. Although many of such tumours are likely to be grade 1 NETs, these do have metastatic potential and careful consideration should be given to the safety of conservative management of appendicitis as a definitive treatment strategy in younger patients.

## Supplementary Material

zrac103_Supplementary_Data

## Data Availability

The data were provided specifically and under licence for this project and are not available for sharing. The data are available from NHS digital via their approval service. We are happy to share the analysis methodology by contacting the corresponding author.
