# Supplementary material for: Demographic trends in the incidence of malignant appendiceal tumours in England between 1995 and 2016: Population-based analysis
Source: BJS Open. 2022 Aug 27;6(4):zrac103. doi: 10.1093/bjsopen/zrac103 (PMC9418812; doi:10.1093/bjsopen/zrac103)
Supplement: zrac103_Supplementary_Data [file zrac103_supplementary_data.docx]

| **Table S1.** Joinpoint segmentation and annual percent change for overall cohort & neuroendocrine tumour cohort | | | | | |
| --- | --- | --- | --- | --- | --- |
| **Cohort** | **Joinpoint Segment** | **Segment years** | **Segment APC (95% c.i.)** | **p-value** | **AAPC (95% c.i.)** |
| **Overall cohort** | | | | |  |
| **Overall incidence** | 1 | 1995 – 2010 | 7.5 (6.6 - 8.4) | < 0.001 | 8.1 (5.1 - 11.2) |
|  | 2 | 2010 – 2013 | 23.0 (1.8 - 48.7) | 0.034 |  |
|  | 3 | 2013 – 2016 | -2.2 (-11.0 - 7.5) | 0.624 |  |
|  | | | | |  |
| **Gender** |  |  |  |  |  |
| Males | 1 | 1995 - 2004 | 3.4 (0.3 - 6.7) | 0.035 | 7.3 (5.6 - 9.1) |
|  | 2 | 2004 - 2016 | 10.3 (8.1 - 12.6) | < 0.001 |  |
| Females | 1 | 1995 - 2016 | 9.3 (8.2 - 10.5) | < 0.001 | 9.3 (8.2 - 10.5) |
|  | | | | |  |
| **Age** |  |  |  |  |  |
| 20-29 | 1 | 1995 - 2016 | 15.6 (12.7 - 18.6) | < 0.001 | 15.6 (12.7 - 18.6) |
| 30-39 | 1 | 1995 - 2016 | 14.2 (12.2 - 16.2) | < 0.001 | 14.2 (12.2 - 16.2) |
| 40-49 | 1 | 1995 - 2016 | 8.0 (6.4 - 9.7) | < 0.001 | 8.0 (6.4 - 9.7) |
| 50-59 | 1 | 1995 - 2003 | 2.5 (-2.3 - 7.5) | 0.290 | 8.1 (5.8 - 10.5) |
|  | 2 | 2003 - 2016 | 11.7 (9.2 - 14.3) | < 0.001 |  |
| 60-69 | 1 | 1995 - 2016 | 9.0 (7.7 - 10.4) | < 0.001 | 9.0 (7.7 - 10.4) |
| 70-79 | 1 | 1995 - 2016 | 6.8 (5.7 - 8.0) | < 0.001 | 6.8 (5.7 - 8.0) |
| 80+ | 1 | 1995 - 2016 | 7.0 (5.3 - 8.8) | < 0.001 | 7.0 (5.3 - 8.8) |
|  | | | | |  |
| **Index of multiple deprivation quintile** |  |  |  |  |  |
| 1 | 1 | 1999 - 2016 | 11.0 (9.1 - 12.9) | < 0.001 | 11.0 (9.1 - 12.9) |
| 2 | 1 | 1999 - 2007 | 6.1 (3.0 - 9.2) | 0.001 | 8.0 (4.7 - 11.3) |
|  | 2 | 2007 - 2012 | 17.2 (7.6 - 27.6) | 0.002 |  |
|  | 3 | 2012 - 2016 | 1.0 (-7.3 - 10.0) | 0.803 |  |
| 3 | 1 | 1999 - 2010 | 7.8 (5.4 - 10.3) | < 0.001 | 8.6 (2.2 - 15.4) |
|  | 2 | 2010 - 2013 | 26.6 (-10.0 - 78.2) | 0.155 |  |
|  | 3 | 2013 - 2016 | -4.6 (-19.6 - 13.2) | 0.553 |  |
| 4 | 1 | 1999 - 2016 | 10.0 (7.7 - 12.5) | < 0.001 | 10.0 (7.7 - 12.5) |
| 5 | 1 | 1999 - 2016 | 11.0 (9.3 - 12.7) | < 0.001 | 11.0 (9.3 - 12.7) |
|  | | | | | |
| **Tumour type** |  |  |  |  |  |
| Neuroendocrine tumour | 1 | 1995 – 2010 | 8.3 (6.9 - 9.7) | < 0.001 | 10.7 (5.8 - 15.8) |
|  | 2 | 2010 – 2013 | 43.4 (5.8 – 94.4) | 0.023 |  |
|  | 3 | 2013 – 2016 | -4.7 (-18.2 – 10.9) | 0.504 |  |
| Adenocarcinoma not otherwise specified | 1 | 1995 - 2016 | 5.8 (4.6 – 7.0) | < 0.001 | 5.8 (4.6 - 7.0) |
| Cystic, mucinous and serous adenocarcinoma | 1 | 1995 - 2009 | 9.0 (7.5 – 10.6) | < 0.001 | 5.3 (3.7 - 7.0) |
|  | 2 | 2009 - 2016 | -1.7 (-5.7 – 2.4) | 0.383 |  |
| **Neuroendocrine tumour cohort** | | | | |  |
| **Overall incidence** | 1 | 1995 - 2010 | 8.3 (6.9 - 9.7) | < 0.001 | 10.7 (5.8 - 15.8) |
|  | 2 | 2010 - 2013 | 43.4 (5.8 - 94.4) | 0.023 |  |
|  | 3 | 2013 - 2016 | -4.7 (-18.2 - 10.9) | 0.504 |  |
|  | | | | |  |
| **Age** |  |  |  |  |  |
| 20-49 | 1 | 1995 - 2010 | 8.8 (6.2 - 11.6) | < 0.001 | 12.4 (3.1 - 22.6) |
|  | 2 | 2010 - 2013 | 56.3 (-13.0 - 180.8) | 0.124 |  |
|  | 3 | 2013 - 2016 | -4.9 (-29.0 - 27.4) | 0.718 |  |
| 50+ | 1 | 1995 - 1998 | 27.6 (10.6 - 47.1) | 0.003 | 11.5 (6.6 - 16.5) |
|  | 2 | 1998 - 2009 | 4.9 (2.6 - 7.3) | 0.001 |  |
|  | 3 | 2009 - 2012 | 36.9 (3.0 - 82.0) | 0.033 |  |
|  | 4 | 2012 - 2016 | 2.0 (-6.8 - 11.6) | 0.644 |  |
|  | | | | |  |
| **Gender** |  |  |  |  |  |
| Male | 1 | 1995 - 2007 | 5.5 (1.8 - 9.4) | 0.006 | 10.5 (7.3 - 13.8) |
|  | 2 | 2007 - 2016 | 17.5 (11.1 - 24.2) | < 0.001 |  |
| Female | 1 | 1995 - 1997 | 43.9 (-8.6 - 126.4) | 0.105 | 14.6 (6.3 - 23.5) |
|  | 2 | 1997 - 2009 | 7.6 (4.4 - 11.0) | < 0.001 |  |
|  | 3 | 2009 - 2012 | 43.8 (-8.6 - 126.3) | 0.106 |  |
|  | 4 | 2012 - 2016 | 3.9 (-10.0 - 20.0) | 0.566 |  |
|  |  |  |  |  |  |
| **Tumour grade** |  |  |  |  |  |
| G1 | 1 | 1995 - 2003 | 4.8 (-12.6 - 25.6) | 0.587 | 34.0 (17.4 - 52.8) |
|  | 2 | 2003 - 2012 | 89.3 (58.0 - 126.9) | < 0.001 |  |
|  | 3 | 2012 - 2016 | 0.5 (-40.5 - 69.9) | 0.984 |  |
| G2 | 1 | 1995 - 2016 | 23.7 (19.4 - 28.2) | < 0.001 | 23.7 (19.4 - 28.2) |
| G3 | 1 | 1995 - 2016 | 24.9 (20.9 – 29.0) | < 0.001 | 24.9 (20.9 - 29.0) |
| G4 | 1 | 1995 - 2016 | 3.1 (-0.4 - 6.6) | 0.078 | 3.1 (-0.4 - 6.6) |
| GX | 1 | 1995 - 2003 | 10.1 (5.6 - 14.8) | < 0.001 | 3.0 (1.1 - 4.9) |
|  | 2 | 2003 - 2016 | -1.2 (-3.1 - 0.8) | 0.234 |  |
| APC = Annual percent change, AAPC = Average annual percent change, c.i. = confidence interval | | | | | |

| **Table S2. Number of appendiceal neoplasms diagnosed across different UK geographical locations** | | | | |
| --- | --- | --- | --- | --- |
|  | **Overall (n = 7056)** | **NETs (n = 3850)** | **Adenocarcinoma NOS (n = 1314)** | **Cystic, mucinous and serous adenocarcinoma (n = 1892)** |
| East of England | 855 (12%) | 446 (12%) | 141 (11%) | 268 (14%) |
| London | 766 (11%) | 435 (11%) | 147 (11%) | 184 (10%) |
| North East | 344 (5%) | 195 (5%) | 59 (4%) | 90 (5%) |
| North West | 929 (13%) | 552 (14%) | 168 (13%) | 209 (11%) |
| South East | 1209 (17%) | 638 (17%) | 244 (19%) | 327 (17%) |
| South West | 898 (13%) | 430 (11%) | 198 (15%) | 270 (14%) |
| West Midlands | 758 (11%) | 438 (11%) | 145 (11%) | 175 (9%) |
| Yorkshire and The Humber | 689 (10%) | 389 (10%) | 118 (9%) | 182 (10%) |
| NETs = Neuroendocrine tumours, NOS = Not otherwise specified | | | | |
